# A Rare Combination: Dengue Fever Complicated With Guillain-Barre Syndrome

**DOI:** 10.7759/cureus.40957

**Published:** 2023-06-25

**Authors:** Chee Siew Lim, Neerusha Kaisbain, Wei Juan Lim

**Affiliations:** 1 Internal Medicine, Hospital Sultanah Aminah, Johor Bahru, MYS; 2 Cardiology, Queen Elizabeth II Hospital, Kota Kinabalu, MYS; 3 Cardiology, National Heart Institute/Institut Jantung Negara Sdn Bhd (IJN), Kuala Lumpur, MYS

**Keywords:** nerve conduction study, neurology, case report, guillain-barre syndrome, dengue fever

## Abstract

Guillain-Barre syndrome (GBS) is an uncommon neurological complication of dengue viral infection. It is more commonly reported with *Campylobacter jejuni*, Epstein-Barr virus, and Cytomegalovirus infection. We report an uncommon case of a 49-year-old man with dengue fever, who developed bilateral lower limb weakness and areflexia on day two of dengue illness. He was diagnosed with GBS as a sequel of dengue infection with the nerve conduction study showing evidence of demyelinating neuropathy. He recovered gradually without immunotherapy and was discharged after a week of hospitalization.

## Introduction

Dengue fever is an arthropod-borne viral infection that is transmitted by female Aedes aegypti or less commonly by Aedes albopictus mosquitoes. It accounts for an estimated 390 million infections worldwide, with the highest global burden of the disease occurring in Asia (~70%) [1]. In 2019, the highest number of cases recorded in Asia were from Bangladesh (101,000), Malaysia (131,000), the Philippines (420,000), and Vietnam (320,000) respectively [2]. There has been an increase in the number of neurological manifestations associated with dengue viral infection reported in recent years [3]. However, the precise incidence rate still remains unclear [3]. The first neurological manifestation of dengue infection was reported in 1976 as an atypical manifestation of dengue fever in Thailand by Sanguansermsri et al. [3]. Subsequently, the most commonly reported neurological complication of dengue infection was encephalopathy and encephalitis, varying from 0.5% to 6.2% [4].

Other neurological complications of dengue infection include acute disseminated encephalomyelitis, optic neuritis, myelitis, and Guillain-Barre syndrome (GBS) [5]. GBS is a rapidly progressive peripheral neuropathy characterized by ascending, symmetrical muscle weakness with reduced or absent deep tendon reflexes [5]. It can involve respiratory muscles. A total of 10-30% of GBS patients may progress to respiratory distress requiring mechanical ventilation [5]. More common antecedent infections reported in GBS were *Campylobacter jejuni*, *Mycoplasma pneumoniae*, *Haemophilus influenzae*, *Cytomegalovirus*, Epstein-Barr virus, and Influenza virus [6]. We would like to report a case of GBS associated with dengue infection.

## Case presentation

A 49-year-old man with underlying diabetes mellitus and dyslipidemia presented with a history of fever, arthralgia, myalgia, and diarrhea of four days duration. He reported no other warning symptoms of dengue, including abdominal pain or bleeding tendencies. However, he complained of bilateral lower limb weakness and numbness which started from day two of fever. He initially described the symptoms as bilateral lower limb heaviness. It progressively worsened over the course of two days to the extent of having difficulty ambulating. He denied urinary or bowel incontinence and shortness of breath. There was no history of other antecedent infections or vaccination.

Upon clinical examination, his vital parameters were stable. He had a good pulse volume and capillary refill time was less than two seconds. Respiratory, cardiovascular, and abdominal examinations were unremarkable. Neurological examination revealed reduced muscle power (3/5) distally and absent deep tendon reflexes over bilateral lower limbs and upper limbs with downgoing plantar reflexes. The power over bilateral upper limbs was normal. Sensory examination of bilateral upper and lower limbs including pain, vibration, proprioception, and temperature was intact. There were no cranial nerves involved. He was still able to ambulate with the aid of a walking stick.

His blood investigation taken on day four of the illness showed thrombocytopenia with a platelet level of 118 x 109/L and leukopenia with a white cell count of 2.3 x 109/L. He had a normal hemoglobin level of 13.5 g/dL and a hematocrit level of 40%. He had mild hyponatremia with sodium level of 133 mmol/L and mild transaminitis with alanine transaminase of 100U/L and aspartate transaminase of 149U/L. Dengue serology IgM was positive and his dengue virus nonstructural protein 1 (NS1) antigen was negative. His bilateral lower limb weakness persisted despite the correction of the hyponatremia. His thyroid function test was normal. A lumbar puncture was not performed as the patient did not consent to the procedure.

A nerve conduction study (NCS) showed bilateral asymmetrical mixed sensory and motor demyelinating polyneuropathy with sural nerve sparing. It predominantly involved the motor nerves. Reduced motor conduction velocity is noted, mostly affecting the lower limb nerves. Prolonged F-wave latencies are seen in all the nerves tested which was consistent with acute inflammatory demyelinating polyneuropathy (AIDP) of bilateral lower limbs (Tables 1-3). A diagnosis of dengue fever complicated with GBS, AIDP variant was made. He was monitored closely for ascending muscle weakness and respiratory involvement. The patient did not receive any immunotherapy as his neurological deficit was over-plateau and showed improvement in lower limb muscle power to 4/5 on day seven of illness in the recovery phase of dengue fever. He underwent physiotherapy, rehabilitation, and occupational therapy.

**Table 1:** Sensory Nerve Conduction Study

Nerve/Sites	Latency (ms)	Pk Amp (µV)	Velocity (m/s)
Left Median - Digit II wrist	4.40	5.1	41.2
Right Median - Digit II wrist	4.00	6.4	43.1
Left Ulnar - Digit V wrist	3.25	15.5	68.3
Right Ulnar - Digit V wrist	3.30	11.2	60.9
Left Sural - Lateral Malleolus Calf	3.50	21.7	51.9
Right Sural - Lateral Malleolus Calf	3.45	19.2	51.9

**Table 2:** F Wave

Nerve	Min F Lat (ms)	Max F Lat (ms)	Mean F Lat (ms)
Left Median Abductor Pollicis Brevis	32.80	33.85	33.08
Left Ulnar Abductor Digiti Minimi	29.30	31.00	30.38
Left Tibial (Knee) Abductor hallucis	56.50	59.90	58.28
Left Common Peroneal Extensor Digitorum Brevis	65.25	66.60	65.84
Right Median Abductor Pollicis Brevis	32.75	34.90	34.04
Right Ulnar Abductor Digiti Minimi	29.55	31.15	30.46
Right Tibial (Knee) Abductor hallucis	60.45	62.00	61.12
Right Common Peroneal Extensor Digitorum Brevis	51.25	82.90	60.61

**Table 3:** Motor Nerve Conduction Study

Nerve/Sites	Latency (ms)	Amplitude (mV)	Velocity (m/s)
Left Median Abductor Pollicis Brevis Wrist	4.45	6.6	
Left Median Abductor Pollicis Brevis Elbow	9.25	6.3	41.7
Right Median Abductor Pollicis Brevis Wrist	4.55	4.9	
Right Median Abductor Pollicis Brevis Elbow	9.75	4.0	38.5
Left Ulnar Abductor Digiti Minimi Wrist	2.95	11.0	
Left Ulnar Abductor Digiti Minimi Below Elbow	6.85	10.3	59.0
Left Ulnar Abductor Digiti Minimi Above Elbow	8.40	10.0	64.5
Right Ulnar Abductor Digiti Minimi Wrist	3.00	9.3	
Right Ulnar Abductor Digiti Minimi Below Elbow	7.15	9.4	55.4
Right Ulnar Abductor Digiti Minimi Above Elbow	8.80	8.8	60.6
Left Tibial (Knee) Abductor Hallucis Ankle	3.95	11.5	
Left Tibial (Knee) Abductor Hallucis Knee	14.05	8.2	34.7
Right Tibial (Knee) Abductor Hallucis Ankle	4.30	10.7	
Right Tibial (Knee) Abductor Hallucis Knee	15.10	7.1	32.4
Left Common Peroneal Extensor Digitorum Brevis Ankle	4.25	2.6	
Left Common Peroneal Extensor Digitorum Brevis Fibula Head	13.90	1.6	35.2
Left Common Peroneal Extensor Digitorum Brevis Knee	15.95	1.5	39.0
Right Common Peroneal Extensor Digitorum Brevis Ankle	4.35	2.1	
Right Common Peroneal Extensor Digitorum Brevis Fibula Head	13.20	1.8	38.4
Right Common Peroneal Extensor Digitorum Brevis Knee	15.25	1.7	39.0

The patient was given intravenous fluid therapy for dengue fever with close monitoring of the neurological deficit. The patient recovered fully from dengue fever and GBS during his follow-up. Full blood count was within normal range with a normal neurological examination.

## Discussion

Dengue fever often affects tropical and subtropical regions around the world, including Malaysia. Dengue virus has four distinct serotypes namely DENV-1, DENV-2, DENV-3, and DENV-4; each of the four can be isolated in Malaysia but usually, a particular serotype will predominate for at least two years before being replaced by another serotype [7]. Initially, dengue neurotropism in human hosts was considered as an opportunistic illness; until 1996 dengue viral antigen was detected in cerebrospinal fluid and central nervous system which proved its neurovirulence [8,9]. DENV-2 and -3 are more commonly associated with neurological complications [10]. Murthy JM has proposed the classification of neuropathogenesis of dengue infection into (1) metabolic disturbances - encephalopathy; (2) direct viral invasion - encephalitis, meningitis, myelitis; and (3) autoimmune reactions - acute disseminated encephalomyelitis, neuromyelitis optica, optic neuritis, myelitis, post-infectious encephalopathy, GBS [4,10].

GBS has different variants which are categorized into AIDP, which is the most common type, acute motor axonal neuropathy (AMAN), acute motor sensory neuropathy (AMSAN), Bickerstaff brainstem encephalitis (BBE) overlap, and Miller Fisher syndrome (MFS) [5,11]. The nerve conduction study of this patient showed reduced conduction velocity, increased latency, and prolonged F wave which was consistent with the AIDP variant of GBS. The pathophysiology of GBS is described as molecular mimicry in *Campylobacter jejuni *infection as it is the most common antecedent infection. It is a cell-mediated immunological response against a bacterial antigen or component that has a structural resemblance to the host neuron which can be targeted to axons or myelin [12]. The inflammatory cytokines produced from the immune reaction towards dengue infection are proposed to have an important role in the pathogenesis of GBS by affecting the permeability of the blood-brain barrier towards the antibody [4,12,13]. Likewise, our patient who developed ascending paralysis on day two of illness suggests that inflammatory cytokines from acute dengue infection have an important role in GBS.

Our patient presented with symmetrical lower limbs weakness on day two of fever (febrile phase) which is relatively uncommon, as most case studies reported the occurrence of GBS after one to two weeks from the onset of dengue illness. A case review of neurological manifestations of dengue infection by Guo et al. reported that the average time it takes for neurological signs of GBS to develop was one to 19 days after the onset of dengue [12]. Another case report by Dalugama et al. in 2018 reported a patient who had lower limb weakness within two days of dengue fever [13]. The development of GBS with evidence of abnormal electrophysiological findings in a proven case of the early phase of dengue suggests that these two diseases are unlikely to be coincidental. There was no respiratory involvement or further ascending paralysis in this patient. The dengue fever complicated with GBS is confirmed by nerve conduction study and positive serology of the dengue-specific IgM. The patient presented with mild disease and thus not requiring immunotherapy. He subsequently improved clinically and was discharged with minimal deficit followed by gradual recovery with rehabilitation.

A meta-analysis of six phases of two trials has shown that removing circulating immune complexes via plasma exchange has shown to improve the recovery time for the ability to walk, the need and duration for ventilation, and measured muscle power after one year as compared to supportive treatment alone [14,15]. When initiated within two weeks of symptom onset, intravenous immunoglobulin has proven to have similar efficacy as plasmapheresis with the advantage of easier administration [16]. However, not all patients require immunotherapy. It is recommended in patients who are nonambulatory and who are within four weeks of neuropathic symptoms and are not showing recovery yet [17]. Our patient was still able to ambulate despite the weakness and he recovered without the need for immunotherapy. The prognosis of GBS is favorable with timely intervention; as 60% of patients achieve full recovery of motor strength. However, there is a mortality rate of approximately 3-7% especially for severe disease and those requiring prolonged ventilation [18,19].

## Conclusions

Dengue fever with GBS is a rare combination and should be kept in mind when encountering patients with limb weakness during dengue fever. Timely diagnosis and treatment for GBS in dengue fever is important and patients should be closely monitored because it may lead to respiratory failure that requires mechanical ventilation. With the surge of dengue infection, awareness of such combination is important to reduce morbidity and mortality.
